# Perinatal Grief and Post-Traumatic Stress Disorder in Pregnancy after Perinatal Loss: A Longitudinal Study Protocol

**DOI:** 10.3390/ijerph18062874

**Published:** 2021-03-11

**Authors:** Eloisa Fernández-Ordoñez, María González-Cano-Caballero, Cristina Guerra-Marmolejo, Eloísa Fernández-Fernández, Marina García-Gámez

**Affiliations:** 1 Department of Nursing, Faculty of Health Sciences, University of Málaga, 29071 Málaga, Spain; eloisafernandez@uma.es (E.F.-O.); magagadue@uma.es (M.G.-G.); 2 Department of Nursing, School of Nursing, Physiotherapy and Podiatry, University of Seville, 41009 Seville, Spain; mgonzalez79@us.es; 3 Agencia Sanitaria Costa del Sol, 29603 Marbella, Spain; eloisa.fernandez.fernandez@juntadeandalucia.es

**Keywords:** perinatal grief, post-traumatic stress disorder, gender differences, pregnancy

## Abstract

Background: Pregnancies that follow perinatal loss are often associated with mental health disorders, which are not usually treated or even identified. Objectives: The main study aim is to identify the prevalence of symptoms of post-traumatic stress disorder and complicated perinatal grief at different stages of pregnancy following a prior gestational loss. Methods: This descriptive longitudinal study will be conducted with a twelve-month follow-up. The study variables addressed will include sociodemographic data (age, sex, education, marital status, employment status and obstetric history) together with clinical data on complicated perinatal grief and post-traumatic stress disorder scores. Results: The results obtained are expected to provide a new perspective on the healthcare approach to perinatal loss and subsequent pregnancy. Conclusions: We seek to optimise comprehensive healthcare in cases of pregnancy following previous perinatal loss and to evaluate options to minimise possible risks.

## 1. Introduction

Perinatal loss can be defined as that which takes place at any time from the moment the pregnancy becomes known until the first month of the child’s life [1]. Consequently, perinatal grief is the parental experience that begins immediately after the loss of a foetus or baby through miscarriage, foetal death, neonatal loss or elective termination due to foetal abnormality [2].

Kennell, Slyter and Klaus (1970) were the first to identify mothers’ reactions to the loss of their newborn and to explore the strength of the bond between the mother and her unborn baby [3]. The acknowledgement of perinatal grief has steadily increased since then, prompted by work such as the 1980 book Motherhood & Mourning: Perinatal Death by Peppers and Knapp [4]. From conception to birth, most parents feel hope, expectation and joy, looking towards the future and not imagining any unhappy outcome [5].

Perinatal loss can be a traumatic experience with significant repercussions that are not only physical but also have psychological and social consequences. Although perinatal loss, especially when it is early, has little social recognition or consideration, a growing body of evidence suggests that its effects can be as shocking as any other loss and should be acknowledged as such [6].

It is estimated that up to 25% of pregnancies end with perinatal loss, and that 50–80% of women who experience perinatal loss conceive again [7]. Accordingly, pregnancy after perinatal loss is a common situation within the clinical practice of obstetrics. Nevertheless, in most cases these women and their families receive no specific care.

The relevance of this work is oriented to the study of the symptoms of perinatal grief and post-traumatic stress disorder (PTSD) in pregnancy after a perinatal loss and the analysis of related risk factors. Although there have been numerous studies on PTSD and perinatal grief after perinatal loss, there are fewer studies that address these symptoms during the course of a subsequent pregnancy and its evolution over time. 

In view of these considerations, the study presented is intended to extend our understanding of the consequences of perinatal loss on the parents’ mental health and of how related problems may arise even after the successful outcome of a subsequent pregnancy. The ultimate treatment goal in this area of healthcare is to improve the status of those involved following a gestational loss and to ensure the necessary resources are available to provide the services needed.

The study aim is to identify the prevalence of symptoms of PTSD and complicated perinatal grief at different stages of pregnancy following a previous gestational loss.

Specific goals:

1. To identify possible differences in the incidence of complicated perinatal grief and PTSD in the different types of gestational loss.
2. To identify risk factors for the development of complicated perinatal grief and PTSD during pregnancy subsequent to gestational loss.
3. To identify possible gender differences in the prevalence of complicated perinatal grief and PTSD during pregnancy after perinatal loss.

## 2. Materials and Methods 

### 2.1. Design

This quantitative study protocol is based on an observational, descriptive and longitudinal design, with a 12-month follow-up. 

### 2.2. Method

#### 2.2.1. Population

The reference population for the study is that of all women in the province of Malaga (Spain), together with their partners, who have become pregnant after suffering a previous perinatal loss. They will be recruited through the pregnancy consultation, which is a health program for low-risk pregnancies included in the National Public Health System of Spain. Pregnant women attend this consultation early in pregnancy. Collecting the clinical history, the obstetric formula will identify those women who have suffered a perinatal loss before [8]. 

#### 2.2.2. Inclusion/Exclusion Criteria

All women and their partners who attend the Pregnancy Programme consultation at any of the health centres in the province of Malaga and meet the following inclusion criteria: of legal age, have suffered a previous perinatal loss (abortion, voluntary termination of pregnancy, interruption due to foetal anomalies, stillbirth or neonatal death), and who agree to take part will be invited to participate in the study.

Persons who do not understand Spanish, the language in which the questionnaires are presented, will be excluded. 

#### 2.2.3. Sample Size

To determine the prevalence of complicated perinatal grief, for a total population of 13,434 women with full-term pregnancy resulting in live birth (Instituto Nacional de Estadística, 2018), with an alpha value of 0.05 and a precision of 8%, the participation of 112 couples is needed to enable a prevalence of 25% to be detected [7].

To determine the prevalence of PTSD, for a total population of 13,434 women with full-term pregnancy resulting in live birth (Instituto Nacional de Estadística, 2018), with an alpha value of 0.05 and a precision of 8%, the participation of 99 couples is needed to enable a prevalence of 21% to be detected [9].

To determine the relationship between complicated perinatal grief and PTSD, the participation of 103 couples is needed to enable a minimum correlation coefficient of 0.60 to be detected, with an alpha value of 0.05, a power (1-beta) of 0.95 and a null correlation hypothesis of r = 0.35.

To achieve these three objectives, we will therefore obtain a sample of 115 couples.

### 2.3. Follow-up Period

During the follow-up period, the participants will be contacted by email, once during each trimester of the pregnancy and once at the end of the puerperium, i.e., ≥40 days after delivery.

### 2.4. Data Collection

The scale proposed in 1989 by Potvin, Lasker and Toedter [10] and adapted into Spanish by Mota et al. in 2011 [11] will be used to measure perinatal grief. This scale is recommended by the grief research manual of the American Psychological Association and has been applied in many studies of perinatal loss. It is a Likert-type scale that consists of 33 statements with five response options. The recommended cut-off score is >40. The validation of the Spanish-language version obtained a Cronbach’s alpha coefficient of 0.95.

The Davidson Trauma Scale [12], presented in 1997 and adapted into Spanish by Julio Bobes in 2015 [13], will be used to assess post-traumatic stress. This scale consists of 17 items and has a recommended cut-off point of >50. The validation of the Spanish-language version obtained a Cronbach’s alpha coefficient of 0.9.

Sociodemographic data will be collected via an ad-hoc questionnaire.

#### 2.4.1. Variables

- Sociodemographic variables: age, sex, education, marital status, employment status.
- Obstetric history: probable date of delivery, number of pregnancies, number of perinatal losses, number of children, type of perinatal loss, gestation week in which the loss occurred.
- Perinatal grief score: according to the Perinatal Grief Scale.
- PTSD score: according to the Davidson Trauma Scale.

#### 2.4.2. Data Collection 

Study data will be obtained by means of self-administered questionnaires, available via forms on an online application.

#### 2.4.3. Data Analysis

Descriptive statistics will be derived by exploratory analysis and frequency distributions by determining the normality of the distribution of variables (using the Kolmogorov–Smirnov test) and by the analysis of asymmetry, kurtosis and histograms.

Bivariate analysis will be performed and differences evaluated using Student’s t and Wilcoxon tests, according to the normality of the distributions. 

Chi-square tests will be performed to determine the relationships between the qualitative variables considered.

The relationship between PTSD and perinatal grief will be determined by calculating Spearman’s correlation coefficient, with the corresponding 95% confidence intervals.

Finally, multivariate logistic regression models will be constructed to evaluate the factors associated with the appearance of perinatal grief and PTSD.

All analyses will be carried out using SPSS 25 (IBM Corporation, Armonk, NY, USA) GPower (Universität Düsseldorf, Dusseldorf, Germany) and Epidat 4.1 software (Epidemiology Service of the General Directorate of Public Health of the Ministry of Health, Santiago de Compostela, Spain).

### 2.5. Ethical Considerations

This study has been approved by two Research Ethics Committees. During its implementation, all applicable standards of good clinical practice and the ethical principles established in the Declaration of Helsinki will be applied at all times. The current regulations on data protection and privacy will be complied with. Signed informed consent will be obtained from all participants. All participant data will be anonymised by assigning an alphanumeric code. The data processing will be for research purposes only. 

## 3. Discussion

Although the question of perinatal loss has drawn increasing attention, the situation of persons experiencing another pregnancy after such a loss is rarely considered in clinical practice [14]. In parallel, while various studies have addressed the impact on maternal mental health of foetal and neonatal death, very few have included the different types of gestational loss [15], including voluntary interruption [16].

In Spain, in particular, little is known about the impact of perinatal loss during subsequent pregnancy. To our knowledge, no systematic interventions in this respect have been conducted, despite the valuable findings reported from studies elsewhere [17,18,19]. The results obtained in the present investigation are expected to highlight this problem and to draw attention to the need for new perspectives in the approach to perinatal loss.

In relation to our objectives, in previous studies we can observe that Callister uses the term "incongruous grief" to refer to gender differences in the experience of perinatal loss, arguing that mothers tend to exhibit longer and more intense periods of grief, and they are more emotionally expressive [20]. Men, on the other hand, can interpret their role as supporting their partner, while limiting the expression of their own feelings [21,22,23]. Although the symptoms related to perinatal loss are very similar between men and women, the manifestations of these feelings are different [23,24]. However, gender differences aside, perinatal loss is a personal experience, unique to each individual [24,25,26].

In agreement with another of the objectives, the available literature classifies perinatal grief as an important predictor of PTSD symptoms [27]. Engelhard reported that PTSD was diagnosed in up to 25% of pregnant women who had suffered perinatal loss in the first month after the event [28]. In a similar study, Horesh recorded a PTSD of 33.3% after perinatal loss. This author concluded that the loss of a pregnancy, regardless of the gestational age in which it occurs, in addition to causing depression, places women at risk of PTSD and observed that up to 25% of women can meet the criteria of PTSD one month later after suffering a perinatal loss. Occasionally, PTSD symptoms appear during a subsequent pregnancy, and 4% of women develop chronic PTSD [29]. Other researchers have identified a 29% lifetime risk of PTSD after perinatal loss and up to a 21% prevalence of PTSD symptoms during the third trimester of gestation after stillbirth. Even one year after the birth of a healthy baby, after a previous perinatal loss, a prevalence of PTSD ranging from 4% to 6% has been reported [9]. Furthermore, the loss of an unplanned pregnancy is associated with the development of PTSD in both men and women [30].

Regarding risk factors, Armstrong, Hutti and Myers (2009) examined depression, anxiety, and post-traumatic stress disorder experienced during pregnancy and the postpartum period in couples who had previously suffered perinatal loss. These authors found significant correlations between depression, post-traumatic stress disorder and anxiety. Greater intensity of grief was associated with higher levels of PTSD [31]. This condition, if left untreated during the perinatal period, can lead to a variety of disorders, including maternal depression, poor prenatal care, prematurity, risky behavior patterns, excessive weight gain, breastfeeding problems and lack of attachment [32]. According to another study, levels of perinatal grief evolve over time, in both men and women after a subsequent successful pregnancy [33]. The association between perinatal loss and increased levels of anxiety and depression during subsequent pregnancies is well documented [15,33,34,35]. Based on similar studies, a reduction in PTSD and complicated grief symptoms is expected over time, as well as a decrease in symptoms of anxiety and depression and symptoms of grief [36,37] and PTSD [9].

### Limitations

The main limitation envisaged concerns the recruitment of participants (i.e., women who are currently pregnant and have previously suffered perinatal loss). This recruitment will have to be carried out in the health centres involved and will require the cooperation of the personnel in each case. Another possible limitation may be due to not considering the mental health history of the participants. 

Furthermore, the longitudinal nature of this study means that a certain percentage of losses to follow-up must be taken into account. Accordingly, in calculating the necessary sample size, an estimated 15% of losses to follow-up are included.

## 4. Conclusions

As the main outcome from this research, we expect to obtain data confirming the existence of significant levels of PTSD and symptoms of complicated perinatal grief during pregnancy after prior gestational loss. We aim to identify the risk factors for these conditions and to determine their possible evolution over time. The overarching goal is to facilitate the adaptation of comprehensive care for persons in this type of situation and, from a psychosocial perspective, evaluate possible healthcare interventions to enhance outcomes and minimise risks.
